# First detection of African swine fever in a swine farm in Taiwan

**DOI:** 10.3389/fvets.2026.1904772

**Published:** 2026-08-21

**Authors:** Fang-Yu Hsu, Kuo-Jung Tsai, Jen-Chieh Chang, Cheng-Hsiang Ni, Hsin-Meng Liu, Hsin-Pei Lu, Ming-Chung Deng, Nien-Nong Lin, Li-Hwa Du, I-Hsuan Huang, Chien-Hui Liao, Ting-Yu Cheng, Andres Perez, Jesper Chia-Hui Hsu, Yu-Liang Huang

**Affiliations:** 1 Veterinary Research Institute, Ministry of Agriculture, Executive Yuan, New Taipei City, Taiwan; 2 Animal and Plant Health Inspection Agency, Ministry of Agriculture, Taipei City, Taiwan; 3 Department of Large Animal Clinical Sciences, College of Veterinary Medicine, Michigan State University, East Lansing, MI, United States; 4 Center for Animal Health and Food Safety, College of Veterinary Medicine, University of Minnesota, Minneapolis, MN, United States

**Keywords:** African swine fever, diagnostic, genotype I/II recombinant, phylogenetic analyses, whole genome sequencing

## Abstract

**Introduction:**

African swine fever (ASF) is a highly contagious, high-consequence transboundary animal disease that poses a critical threat to global swine production and agricultural economics. Since its emergence in China in 2018, ASF has spread to over 20 Asia-Pacific countries, causing significant economic disruption. While Taiwan previously detected ASF virus several times in dead pigs drifting offshore, or in illegal pork-related products brought by international tourists and inspected at airport and seaport border controls, no local domestic swine farm had tested positive before this October 2025 ASF event. However, maintaining this disease-free status requires constant vigilance against evolving regional biosecurity threats.

**Methods:**

The first ASF detection in a domestic swine herd in Taichung City, Taiwan, reported on October 22, 2025, was triggered by abnormal alerts of the monitoring system in the rendering plant; the index farm captured a cumulative herd mortality rate of 35.2% that exceeded the predefined threshold (3% mortality daily in the nursery to finish pigs). Five finisher pigs were submitted for diagnostic evaluation, which subsequently confirmed ASFV infection via real-time PCR, pathological examination, immunohistochemistry, virus isolation, and whole-genome sequencing.

**Results:**

Affected pigs showed clinical signs including wheezing, sudden death, nasal bleeding, uncoagulated blood in the nostrils, and mild hemorrhage on the skin surface of the neck, abdomen, and buttocks. Histopathological examination revealed severe multisystemic hemorrhagic lesions. Based on assay results of the P72, P54, P30, and CD2v genes, and whole-genome sequence, phylogenetic analysis confirmed that the isolate (ASFV/TWN/2025) is a genotype I/II recombinant strain, most similar to prevailing strains isolated in China and Vietnam, sharing 99.95%–99.97% and 99.92%–99.97% nucleotide similarity of whole genome sequence, respectively.

**Discussion:**

Upon confirmation of the ASF case, authorities immediately implemented a nationwide swine movement standstill for 15 days to mitigate transmission risk. To date, no secondary cases have been detected. This article details the early monitoring and rapid diagnosis process of the first case of ASF infection in a farm in Taiwan, and highlights the information from this case to provide lessons for disease diagnosis and prevention in ASF-free areas.

## Introduction

African Swine Fever (ASF) is a highly contagious, viral hemorrhagic disease with high mortality in domestic (*Sus domesticus*) and wild swine. The clinical signs of acute infection include hemorrhage, fever, decreased appetite, vomiting, abortion, and sudden death. The virus is stable in the environment and remains infectious for weeks to months in frozen or uncooked meat (1), and has spread transboundary into 20 countries in the Asia Pacific, and other continents (e.g., Africa, Europe, and the Caribbean Americas) primarily through human activities. As a major threat to the global swine industry (2), the World Organisation for Animal Health (WOAH) has identified ASF as a notifiable disease (3).

ASF is caused by the ASF virus (ASFV), a large, double-stranded DNA virus that belongs to the *Asfarviridae* family (1, 3). The ASFV genome is a complex, linear DNA molecule of 170–190 kb, encoding a variety of structural and non-structural proteins. Based on its P72 capsid structure protein, ASFV can be classified into 24 genotypes (1, 3). The strains circulating in the Asia Pacific were predominantly genotype II, originating from the Georgia 2007/1 strain that expanded eastward into China and the Asia-Pacific region. After 2021, diverse ASFV strains have been reported, including genotype I, genotype II with gene deletions, and genotype I/II recombinant strains (rASFV) (3–5). Although live attenuated vaccines (LAVs) for genotype II have been developed and commercialized in Vietnam (6) and reported in other countries, such as China (7). Consequently, the risk of shedding vaccine strains, restoring virulence, and recombining with wild viruses remain a concern in the scientific community, as advised by WOAH (8, 9).

Taiwan comprises four primary island groups, namely, the main island of Taiwan, and the archipelagos of Penghu, Kinmen, and Matsu. The main island is the largest, accounting for approximately 98% of the total land area. As of 2025, there were approximately 5,495 swine farms in Taiwan, with the main island accounting for 99% of swine production facilities and the total pig population (i.e., approximately 5.3 million head of pigs). Additionally, native Taiwanese wild boars are present, though they are primarily distributed in mountainous regions in relatively low numbers.

Due to its insular geography, Taiwan has possessed inherent advantages for the control of transboundary swine diseases, such as ASF. For years, the main island successfully maintained its ASF-free status through the implementation of stringent biosecurity protocols. These measures included rigorous border surveillance at major ports of entry (10) and veterinary inspection of both passenger luggage and commercial cargo. To deter the illegal importation of pork products from ASF-affected regions, Taiwan enforces a substantial penalty, fining individuals NTD 200,000 (USD 7,000) for a first-time offense and NTD 1,000,000 (USD 35,000) for subsequent violations (11). Consequently, while viral DNA was periodically detected in pig carcasses washing ashore on outlying islands (most frequently in the Kinmen and Matsu islands), the domestic swine industry on the main island remained unaffected (12).

Nevertheless, in 2025, ASFV was reported in domestic pigs on the main island, representing a significant breach in biosecurity defenses and posing a severe threat to both the national economy and the stability of the swine industry across East and Southeast Asia. The outbreak was successfully contained to one production facility, leading to the restoration of ASF-free status from WOAH on April 6, 2026 (13) This study aims to document the first ASFV case in domestic swine and share the integrating clinical observation, pathological findings, epidemiological tracing, and molecular confirmation with an analysis of phylogenetic characterization, with the scientific community.

## Materials and methods

### Case farm background

**Figure 1:**
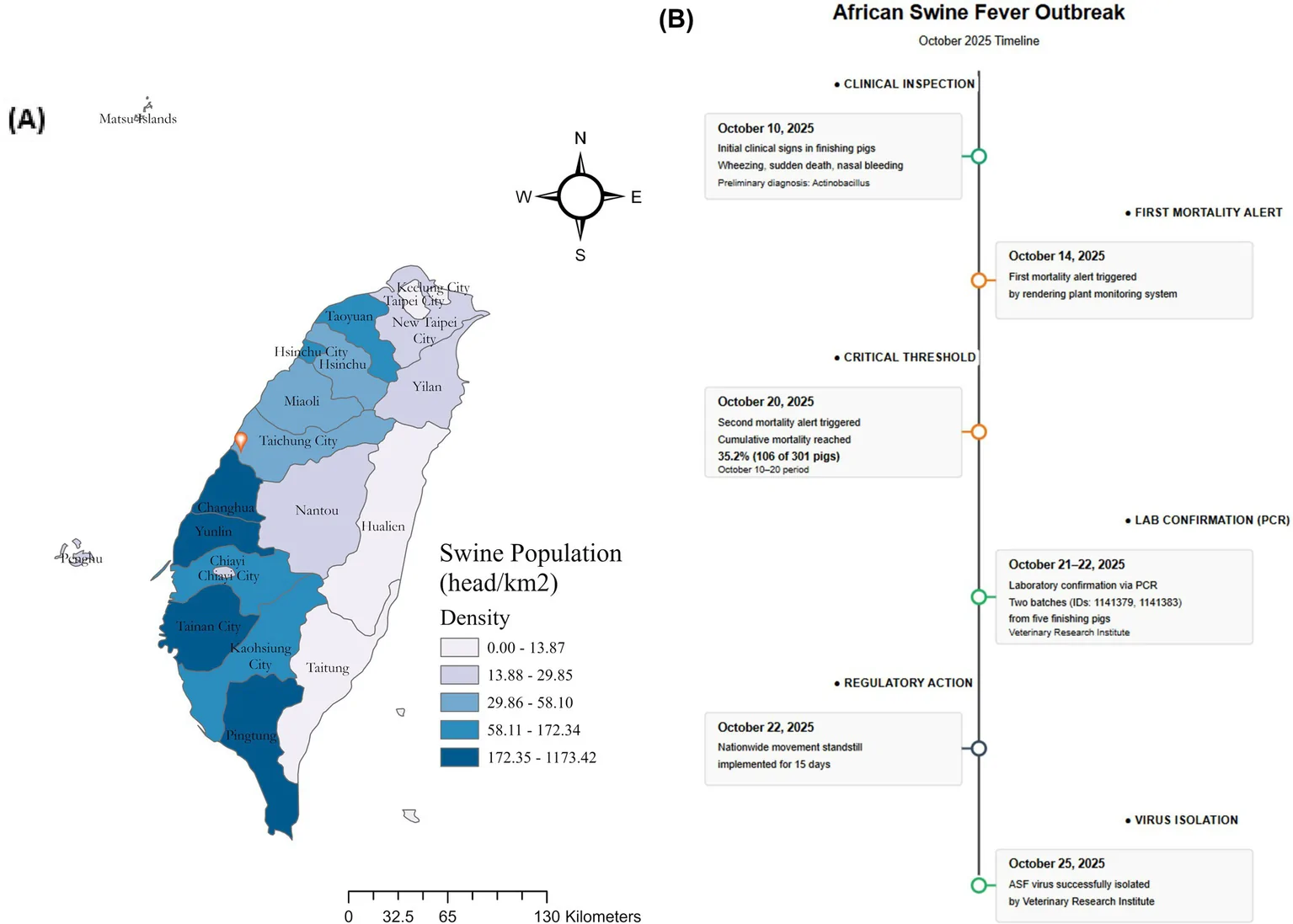
**(A)** Spatial distribution of swine density and the ASF first outbreak in Taiwan. Darker blue shading indicates higher swine density (heads/km^2^), concentrated in central-western and southern counties. The red marker identifies the ASF outbreak farm situated in Wuqi District, Taichung City. **(B)** Timeline of the outbreak, including initial clinical suspicion, mortality alerts triggered by the rendering plant monitoring system, laboratory diagnosis, and implementation of regulatory control measures.

The case farm was located in Wuqi District, Taichung City, Taiwan (Figure 1A). The farm was a traditional continuous farrow-to-finish operation housing 301 pigs, which were fed on uncooked food waste and commercial feed. Clinical signs, including wheezing, sudden death, and nasal bleeding, were first observed in finishing pigs on October 10, 2025 (Figure 1B). The herd veterinarian conducted a clinical inspection, and a preliminary diagnosis suggested a bacterial infection, with *Actinobacillus pleuropneumoniae* as the suspected cause. A sudden increase in mortality served as an early warning for the national mortality monitoring system of rendering plants on October 14 and again on October 20. Between the clinical inspection (Oct. 10) and the mortality alert (Oct. 20), a total of 106 pigs died, representing a mortality rate of 35.2% (106/301). On October 21 and 22, 2025, two batches (ID: 1141379 and 1141383) of samples were collected from two (ID: 1141379–1 and 1141379–2) and three (ID: 1141383–1, 1141383–2, and 1141383–3) finisher pigs by the Taichung Local Animal Disease Inspection Authority and submitted to the Veterinary Research Institute of the Ministry of Agriculture for diagnostic testing.

### Pathological examination and ASFV immunochemistry

The tissues from three pigs (ID: 1141383) in the ASF-affected farm, including spleen, liver, kidney, lung, and lymph nodes, were sampled and fixed in 10% neutral buffered formalin for histopathological examination and ASFV immunohistochemistry (IHC), performed by VRI. Fixed tissues were routinely processed, embedded in paraffin, sectioned at a thickness of 4–5 μm, and mounted on glass slides. The sections were stained with hematoxylin and eosin (H&E) for histopathological examination. Microscopic lesions were evaluated using a light microscope and scored based on the severity and distribution of pathological changes (14).

The ASFV IHC was determined by Super Sensitive™ Polymer-HRP IHC Detection (BioGenex, United States) using anti-ASFV P30 monoclonal antibody, developed by the VRI. Briefly, formalin-fixed tissues were deparaffinized in xylene and rehydrated through a graded series of ethanol. Antigen retrieval was performed by heating the sections in citrate buffer. Endogenous peroxidase activity was blocked using 3% hydrogen peroxide at room temperature for 10 min and Universal Blocking Reagent (BioGenex, United States) at room temperature for 10 min. The sections were then incubated with an anti-ASFV P30 monoclonal antibody at room temperature for 1 h. After washing with Tris-Buffered Saline and Tween 20, the slides were incubated with Poly-HRP (BioGenex, United States) at room temperature for 40 min. Immunoreactivity was visualized using Romulin AEC Chromogen Kit (Biocare, United States) as the chromogen, followed by counterstaining with hematoxylin. Positive staining was identified under a light microscope as brown-colored signals within the cytoplasm or nucleus of infected cells.

### Molecular diagnosis

Nucleic acids were extracted using a commercial viral DNA extraction kit (Taiwan Advanced Nanotech Inc., Taoyuan City, Taiwan) and an automated extraction instrument (Maelstrom 4,810, Taiwan Advanced Nanotech Inc.). Reverse-transcription quantitative PCR (RT-qPCR) was conducted for the detection of classical swine fever virus (CSFV) and porcine reproductive and respiratory syndrome virus (PRRSV), and quantitative PCR (qPCR) was performed for the detection of ASFV, and type 2 porcine circovirus (PCV2) according to the terrestrial animal health manual of WOAH and Chang et al., study (15–17). The primers and probes were summarized in Table 1. The ASFV load in the tissue samples was calculated based on a standard curve established using an ASFV plasmid. Samples positive for ASFV were further subtyped by detecting the deletion of the MGF, CD2v, and I177 gene (Table 1). In addition, conventional PCR assays targeting the P72 gene, including the published P72 D/U primer set (18) and an additional P72 primer set (PPA outer) designed in this study, as well as the B602L gene (central variable region, CVR) (19). PCR positivity was determined at Ct ≤ 40 in the RT-qPCR and qPCR.

**Table 1:** The sequences of primers and probes used in the molecular diagnosis of African swine fever virus (ASFV), classic swine fever virus (CSFV), porcine reproductive and respiratory syndrome virus, and porcine circovirus type 2.

Primer and probe name	5′–3′	References
Forward primer of ASFV-WOAH qPCR	5′-CTGCTCATGGTATCAATCTTATCGA-3′	(16)
Reverse primer of ASFV-WOAH qPCR	5′-GATACCACAAGATCRGCCGT-3′	
Probe of ASFV-WOAH qPCR	5′-FAM-CCACGGGAGGAATACCAACCCAGTG-BHQ-3′	
Forward primer of ASFV-MGF qPCR	5′-GGGTTCGGATACAGGCGTTA-3′	(37)
Reverse primer of ASFV-MGF qPCR	5′-CGTGTTCCTGCCGTGTATCTAA-3′	
Probe of ASFV-MGF qPCR	5′-FAM-CCTCCCAGTTCCGCACACAGCC-BHQ-3′	
Forward primer of ASFV-CD2v qPCR	5′-GAAGAACAATGTCAGCATGATGA-3′	(37)
Reverse primer of ASFV-CD2v qPCR	5′-ACTGTAAGGCTTAGGAAGTAATGGTT-3′	
Probe of ASFV-CD2v qPCR	5′-YAK-ACCACTTCCATACATGAACCATCTCCCA-BHQ-3′	
Forward primer of ASFV-I177L qPCR	5′-GAACTGGAAAAAACTTTAACGGC-3′	(38)
Reverse primer of ASFV-I177L qPCR	5′-CCATTACCGGCAAGCTAGG-3′	
Probe of ASFV-I177L qPCR	5′-FAM-ACGGATCCCCCTTCGCATTTGA-MGB-NFQ-3′	
Forward primer of CSFV RT-qPCR	5′-ATGCCCAYAGTAGGACTAGCA-3′	(17)
Reverse primer of CSFV RT-qPCR	5′-CTACTGACGACTGTCCTGTAC-3′	
Probe of CSFV RT-qPCR	5′-FAM-TGGCGAGCTCCCTGGGTGGTCTAAGT-BHQ-3′	
Forward primer of PRRSV RT-qPCR	5′-ATRATGRGCTGGCATTC-3	(15)
Reverse primer of PRRSV RT-qPCR	5′-ACACGGTCGCCCTAATTG-3′	
Probe of PRRSV RT-qPCR	5′-FAM-TGTGGTGAATGGCACTGATTGACA-BHQ1-3′	
Forward primer of PCV2 qPCR	5′-GCTGGCTGAACTTTTGAAA-3′	(15)
Reverse primer of PCV2 qPCR	5′-CCTTTAGTCTCTACAGTCAATGGAT-3	
Probe of PCV2 qPCR	5′-FAM-TGCTAATTTTGCAGACCCGGAAACCAC-BHQ1-3′	

### ASFV isolation

Porcine alveolar macrophages (PAMs) were rapidly thawed from liquid nitrogen using a 37 °C water bath. The cells were incubated in 24-well plates with Roswell Park Memorial Institute (RPMI) medium supplemented with 10% fetal bovine serum (FBS) at 37 °C with 5% CO_2_ for 24 h prior to inoculation. For ASFV isolation, 100 μL of 0.45 μm-filtered lymphoid tissues (spleen and lymph nodes) was inoculated into each well containing PAMs. After incubation for 18 h at 37 °C with 5% CO_2_, 100 μL of 0.75% washed red blood cells was added to each well. At least one uninoculated well was maintained as a negative control to monitor non-specific hemadsorption (HAD). The plates were further incubated for 7 days at 37 °C with 5% CO_2_ and examined daily for the presence of HAD.

### ASFV whole genome sequencing

Total nucleic acids from isolates 1141383–2 and 1141383–3 were extracted using a commercial extraction kit, and the whole-genome sequencing was performed on the MiSeq System using a MiSeq Reagent Kit. The filtered reads were mapped to reference ASFV genomes available in the GenBank database using a read alignment program. In addition, *de novo* assembly was performed to reconstruct the viral genome. The assembled contigs were further analyzed and curated to obtain complete or near-complete ASFV genome sequences.

### Reference sequences

ASFV reference sequences were selected from isolates recently published on the NCBI GenBank. Sequences of X vaccines were included in the sequencing analysis.

### Phylogenetic and recombination analyses

The phylogenetic analysis was conducted using the MEGA software (version 11) (20, 21). In particular, the Clustal W method was used to align the sequences of the whole genome sequence, P72, P54, P30, and CD2v genes. A phylogenetic tree was generated using the maximum-likelihood method with 1,000 bootstrap replicates under the Tamura 3-parameter or Kimura 2-parameter substitution model (21, 22). The recombination analysis between genotype I and II was performed by SimPlot software 3.5.1 using the Kimura 2-parameter.

### Statistical analysis

Student’s *t*-test and one-way analysis of variance (ANOVA) were, respectively, using for two groups and among multiple groups and were conducted with the Statistical Analysis System (SAS for Windows 9.4; SAS Institute Inc., Cary, NC, United States). A *p*-value of less than 0.05 was considered statistically significant.

## Results

### Gross lesions of ASF-infected pigs

**Figure 2:**
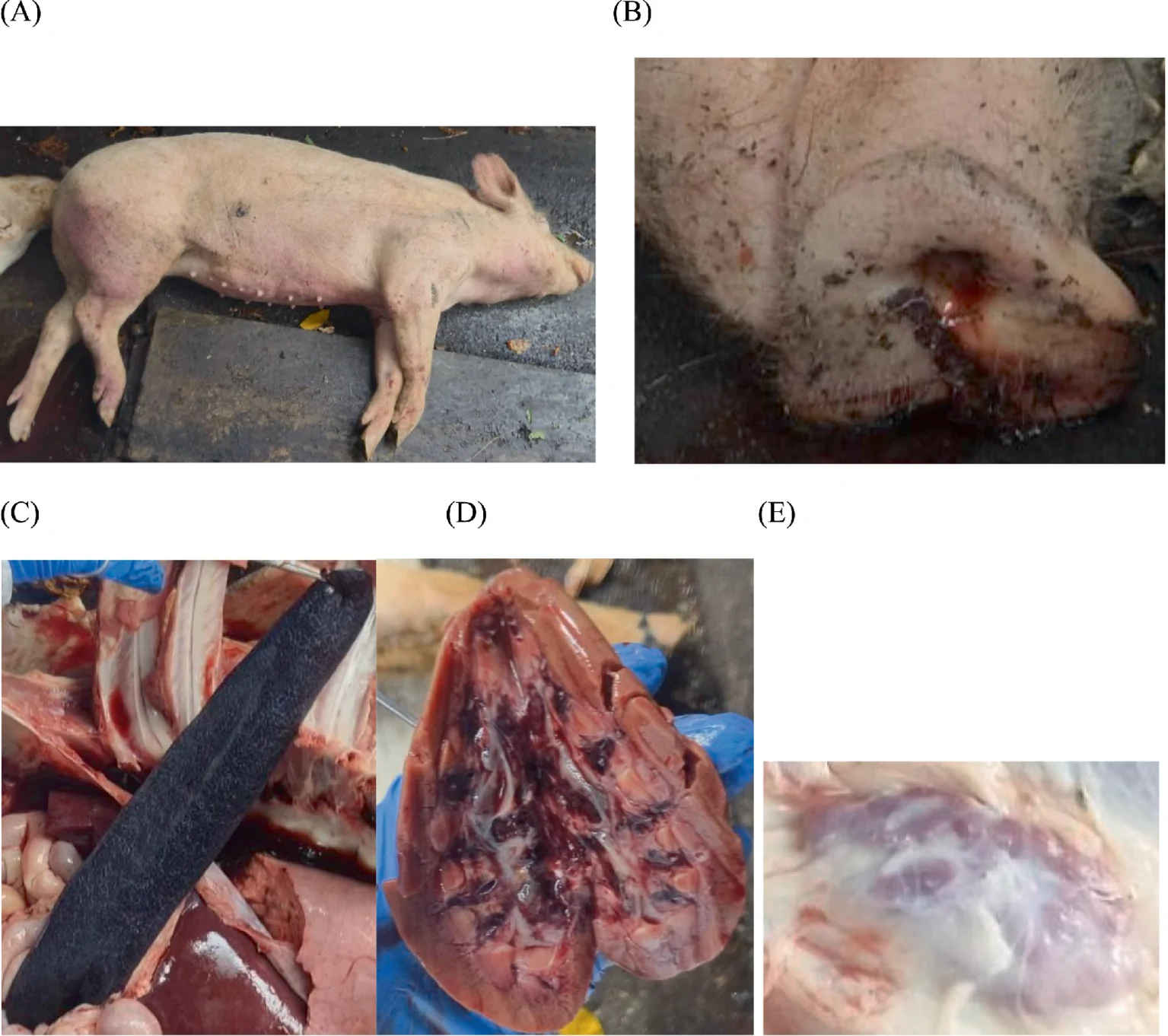
Gross lesions of affected pigs from the case farm. Pathological changes include mild cutaneous hemorrhage on the skin surface of the neck, abdomen, and buttocks **(A)**; uncoagulated blood in the nostrils **(B)**; moderate splenomegaly **(C)**; diffuse hemorrhagic spots on the kidneys **(D)**; and hemorrhage and enlargement of the inguinal lymph nodes **(E)**.

The affected pig showed uncoagulated blood in the nostrils and mild hemorrhage on the skin surface of the neck, abdomen, and buttocks (Figure 2). Necropsy revealed mild to severe hemorrhage and enlargement of the inguinal lymph nodes, diffuse hemorrhagic spots on the cortex and renal pelvis of the kidneys, and moderate splenomegaly.

### Histopathology and IHC examination of infected-cases

**Figure 3:**
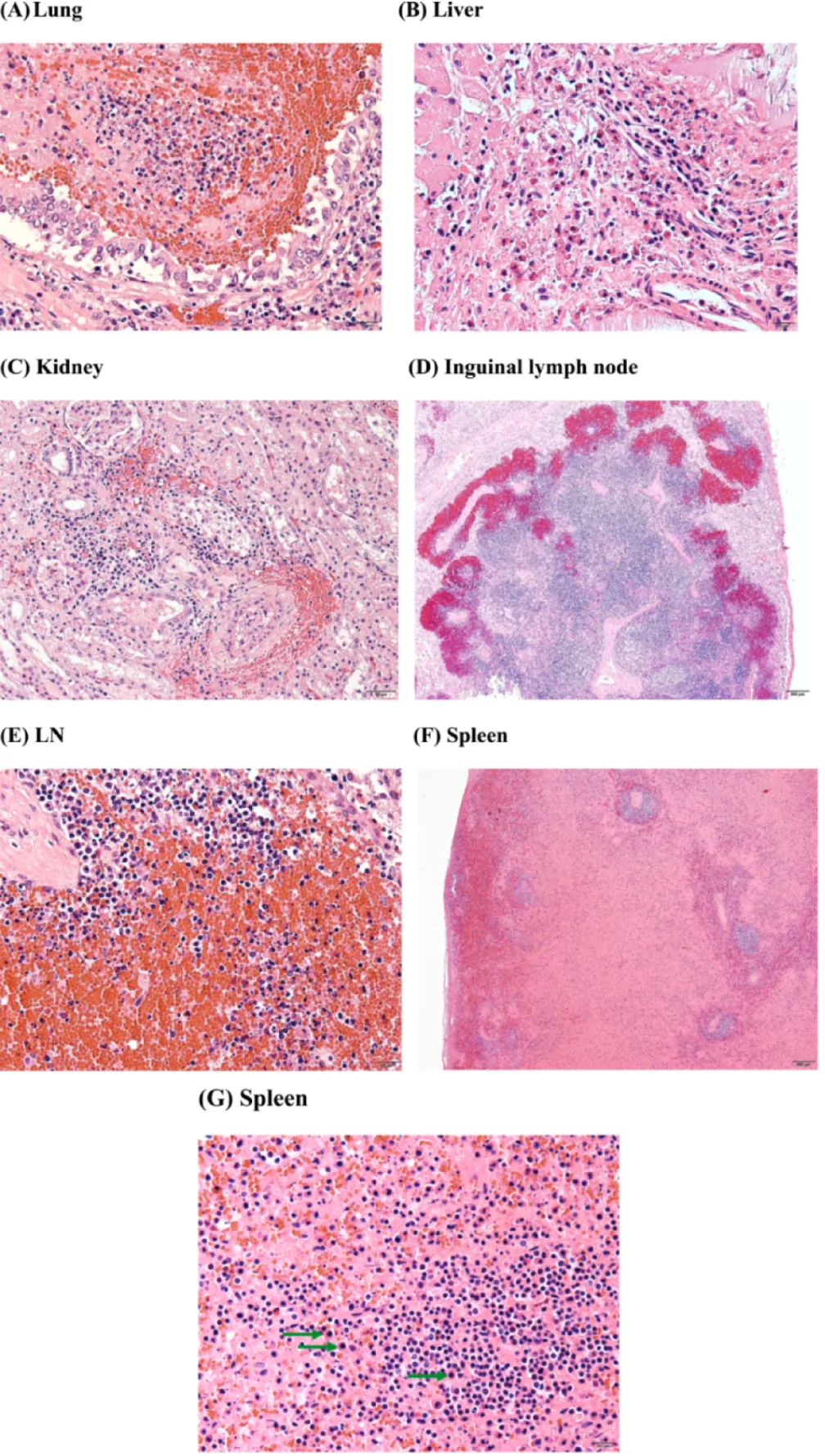
Histopathological examination showing severe multisystemic lesions consistent with hemorrhage and necrosis. **(A–G)** Lesions were observed in the lung (**A**, ×200), liver (**B**, ×400), kidney (**C**, ×200), and inguinal lymph node (**D**, ×100; **E**, ×400). The lymphocytic and eosinophilic portal triaditis was found (**B**, ×400). Lymphocyte necrosis and depletion were observed in the spleen (**F**, ×100; **G**, ×400). Nucleated red blood cells (nRBCs; green arrows) were also identified in the spleen, indicating induced extramedullary hematopoiesis.

Histopathological examination revealed severe multisystemic lesions consistent with acute hemorrhagic disease (Figure 3). In the lungs, severe diffuse subacute hemorrhagic and pulmonary edema was observed with histiocytic interstitial pneumonia. In addition, locally extensive subacute hemorrhagic and suppurative bronchopneumonia with marked peribronchiolar lymphoid hyperplasia were present. The inguinal lymph nodes exhibited acute, severe, and diffusely hemorrhagic and necrotizing lymphadenitis characterized by necrosis of lymphoid follicles with marked lymphoid depletion and extensive lymphocyte apoptosis. The spleen showed severe diffuse congestion and hemorrhage with prominent lymphoid depletion, lymphocyte apoptosis, numerous tangible body macrophages, and increased numbers of nucleated erythrocytes indicative of extramedullary hematopoiesis. The liver demonstrated multifocal mild to moderate chronic lymphocytic and eosinophilic portal triaditis, consistent with parasite infection.

**Figure 4:**
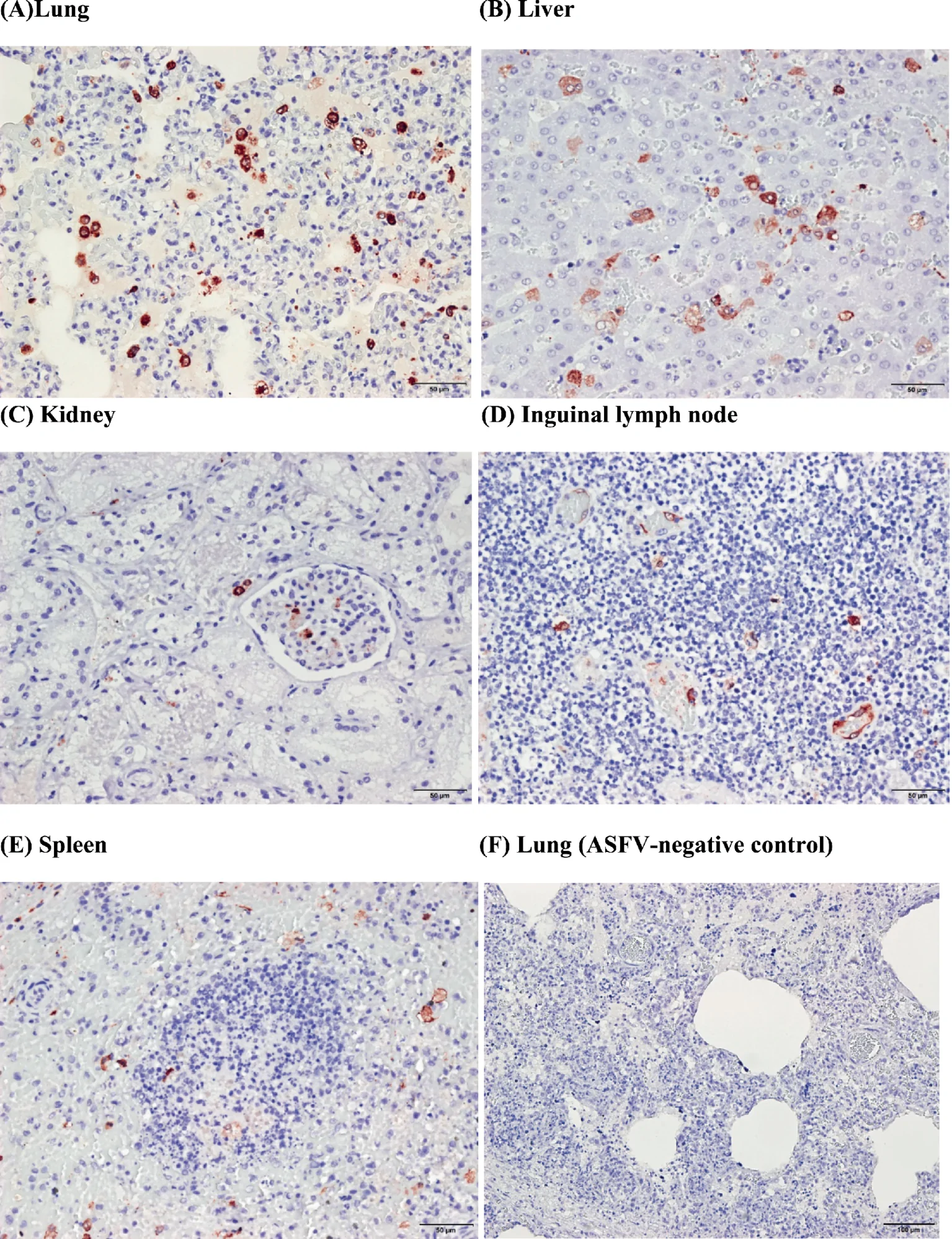
Detection of ASFV antigens by immunohistochemical (IHC) examination using a P30 monoclonal antibody (mAb). **(A–E)** ASFV antigens (brown) were primarily localized within macrophages of the lung (**A**, ×400), liver (**B**, ×400), kidney (**C**, ×400), inguinal lymph node (**D**, ×400), and spleen (**E**, ×400). Lung (**F**, 200×) from ASFV-negative pigs was negative control for ASFV IHC. In addition, partial positivity was also observed in vascular endothelial cells, hepatocytes, and renal tubular epithelial cells.

The IHC examination showed ASFV antigens mainly in macrophages of lymph nodes, spleen, and lung, with only occasional positivity in vascular endothelial cells, hepatocytes, and renal tubular epithelial cells, consistent with systemic dissemination with marked tropism for the mononuclear phagocyte (Figure 4).

### Molecular diagnosis

All samples (*n* = 5) from cases 1141379 and 1141383 were screened positive for the ASFV (Table 2), with the viral loads ranging between 10^5.62^ and 10^6.31^ copies/g in lymph nodes, spleen, kidney, lung, and liver (Table 3). The ASFV loads in the difference tissues were not significant difference. The confirmed ASFV PCRs of ASFV-P72 D/U, PPA outer, and B602L were all positive. Deletions in the region of MGF, CD2v, and I177L, were not detected, suggesting that the isolated ASFV was not a vaccine strain. Additionally, all samples were negative for the detection of CSFV, whereas one pig (1141383–3) tested positive for PRRSV and three pigs (1141379–2, 1141383–1, and 1141383–3) tested positive for PCV2. The PRRSV load in pig 1141383–3 was low in lung and lymph node, range between 10^0.04^ and 10^0.003^TCID_50_/g. The high PCV2 load was found in the lymph node of pigs 1141383–3 with 10^5.5^ PCV2 copy/g. The PCV2 loads in the lymph node of pigs 1141379–2 and 1141383–1 were lower than 10^2.3^ PCV2 copy/g. To compare the ASFV loads between PCV2 infection and non-infection pigs, there were not significant difference.

**Table 2:** The samples of infected cases were determined by molecular diagnosis.

Methods	1141379–1	1141379–2	1141383–1	1141383–2	1141383–3
ASFV-WOAH qPCR	+	+	+	+	+
CSFV RT-qPCR	−	−	−	−	−
PRRV RT-qPCR	−	−	−	−	+
PCV2 qPCR	−	+	+	−	+
ASFV-MGF qPCR	+	+	+	+	+
ASFV-CD2v qPCR	+	+	+	+	+
ASFV-I177L qPCR	+	+	+	+	+
ASFV-P72 D/U PCR	+	+	+	+	+
ASFV-PPA outer PCR	+	+	+	+	+
ASFV-B602L PCR	+	+	+	+	+

**Table 3:** ASFV loads in various tissues were determined by ASFV-WOAH qPCR.

Pigs	Spleen	Lymph nodes	Lung	Liver	Kidney
1,141,379–1	6.20	6.18	5.75	5.86	5.91
1,141,379–2	5.95	5.97	5.67	5.68	5.65
1,141,383–1	6.64	6.05	6.23	6.49	5.10
1,141,383–2	5.83	5.37	4.86	6.87	4.86
1,141,383–3	5.63	6.06	5.80	6.67	6.58
Mean ± SD	6.05 ± 0.35^a^	5.92 ± 0.29^a^	5.66 ± 0.45^a^	6.31 ± 0.46^a^	5.62 ± 0.61^a^

The units of ASFV loads were copy (log)/g. SD is the standard deviations. ^a^No significant differences were observed among tissues.

### ASFV isolation

**Figure 5:**
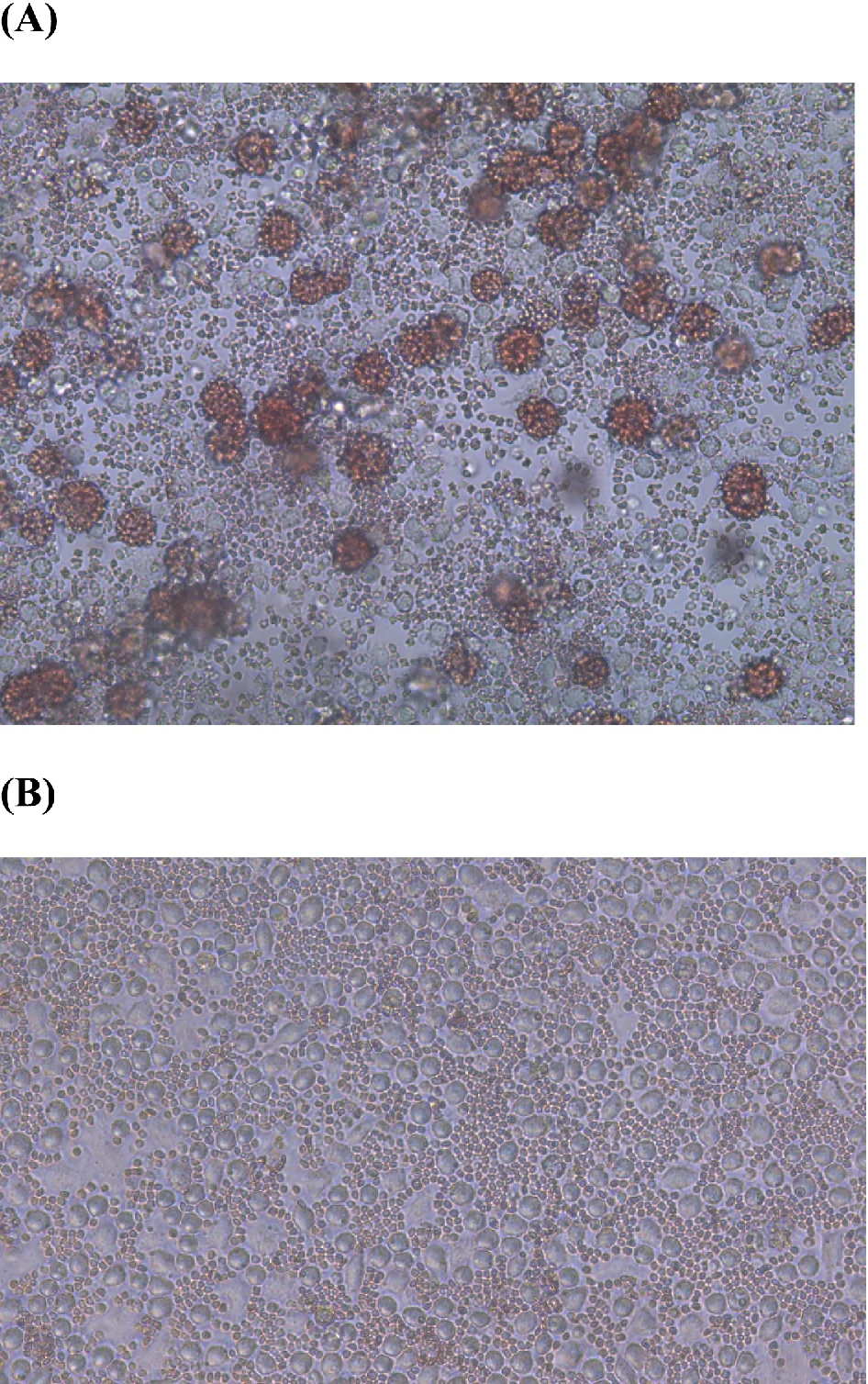
Isolation of ASFV from all pigs was using primary alveolar cells. **(A)** Hemadsorption was observed in all pigs at 3 days post-inoculation. **(B)** Negative control of ASFV isolation was not hemadsorption.

The HAD were observed in PAM inoculated with the lymphoid tissues of all pigs at 3 days post-inoculation (Figure 5). The titer of ASFV isolates ranged between 10^5.45^ and 10^6.1^ HAD_50_/ml. Theses ASF isolates were negative by PRRSV RT-qPCR and PCV2 qPCR.

### Phylogenetic analysis of the whole ASFV genome, and P72, P54, P30, and CD2v genes

**Figure 6:**
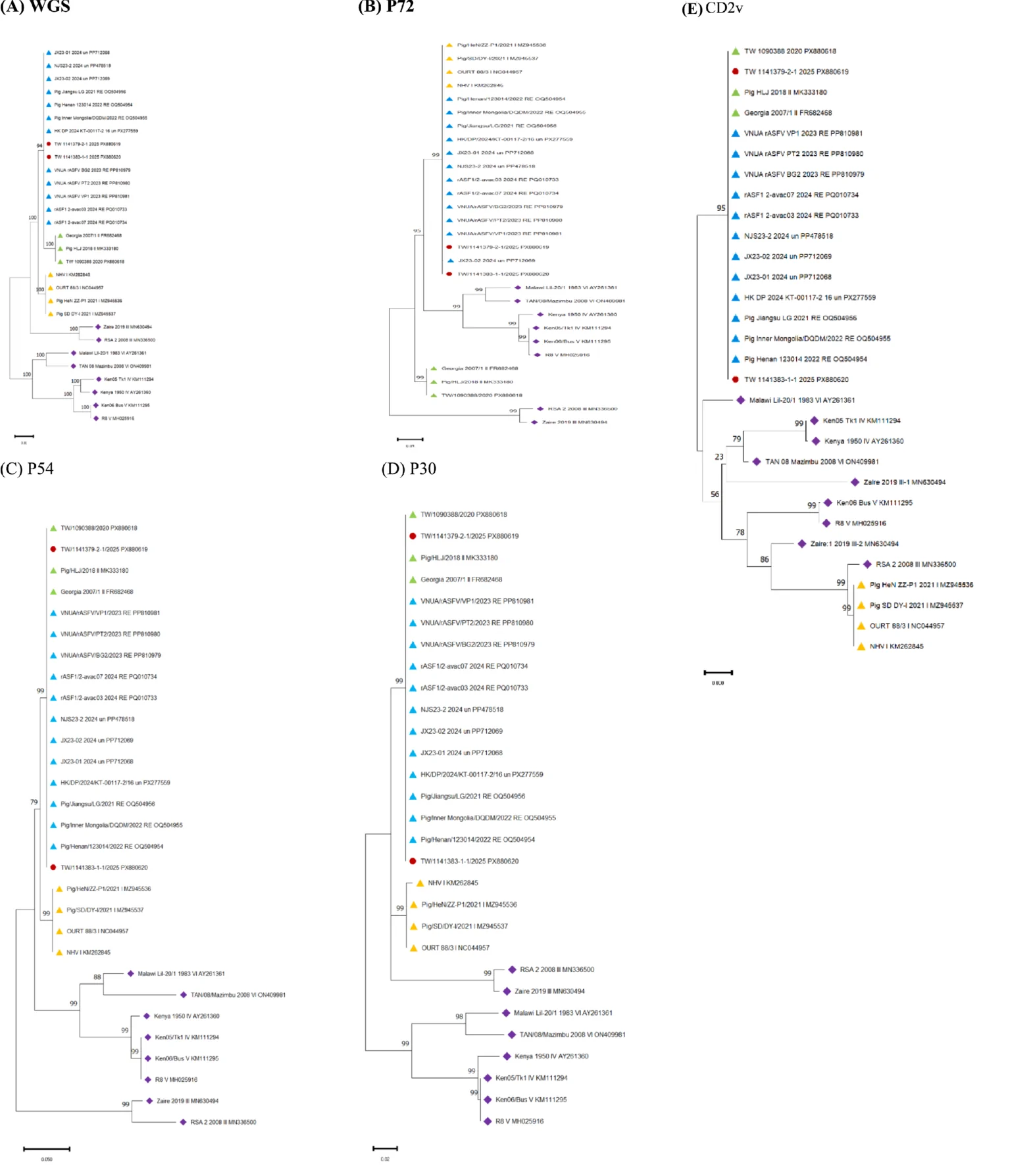
Phylogenetic trees of the whole genome sequence **(A)**, P72 **(B)**, P54 **(C)**, P30 **(D)** and CD2v **(E)** genes in cases 1141379-2-1 (GenBank: PX880619) and 1141383-1-1 (GenBank: PX880620) were constructed using the maximum-likelihood method with 1,000 bootstrapreplicates under the HYK substitution model in MEGA 11. ● indicated the affected domestic swine farm; ▲ indicated recombinant I/II ASFV strain; ▲ indicated genotype I ASFV strain; ▲ indicated genotype II ASFV strain; ◆ indicated non-genotype I and II ASFV strain.

The whole genomes of 1141379–2-1 and 1141383–1-1 isolates were sequenced with the sizes of 187,583 and 188,808 bp, respectively, and have been published on the NCBI GenBank (GenBank Access Number: PX880619 and PX880620). The 1,225 bp difference between 1141379–2-1 and 1141383–1-1 isolates was located in the 5’-NTR and 3’-NTR. Compared to 1141383–2 isolate, and 1141379–2-1 isolate was additional 16 and 21 DRT-34 repeats in the 5’-NTR and 3’-NTR, respectively. The sequence length of function ORFs between 1141379–2-1 and 1141383–1-1 isolates was same and was also same to rASFV/China or rASFV/Vietnam isolates. After BLASTing with the sequences of ASFV reference strains on NCBI GenBank, the percentage identity between the whole genome sequences of cases 1141379–2-1 and 1141383–1-1 and rASFV strains isolated in China and Vietnam ranged between 99.95 and 99.97% and between 99.92 and 99.97%, respectively. To compare the similar distance between genotype I and ASFV/TWN/2025 and between genotype II and ASFV/TWN/2025, the similarity was, respectively, ranged from 98.61 to 98.62% and ranged from 98.73 to 98.74%. This suggests that the isolates are likely to be a rASFV. This was further confirmed by the phylogenetic analysis, where the isolates were classified into rASFV (Figure 6A). Phylogenetic analysis of the P72 gene revealed that the virus isolated from both pigs were classified to genotype I and shared 100% similarity with strains OURT-88-3 (GenBank: NC044957) and HK-DP-2024-KT-00117-2-16 (GenBank: PX277559) (Figure 6B). Conversely, the analysis of the P54, P30, and CD2v genes classified these isolates into genotype II, showing 100% similarity with strains Georgia/2007 (GenBank: FR682468) and HK-DP-2024-KT-001 17-2-16 (GenBank: PX277559) strains (Figures 6C–E).

**Figure 7:**
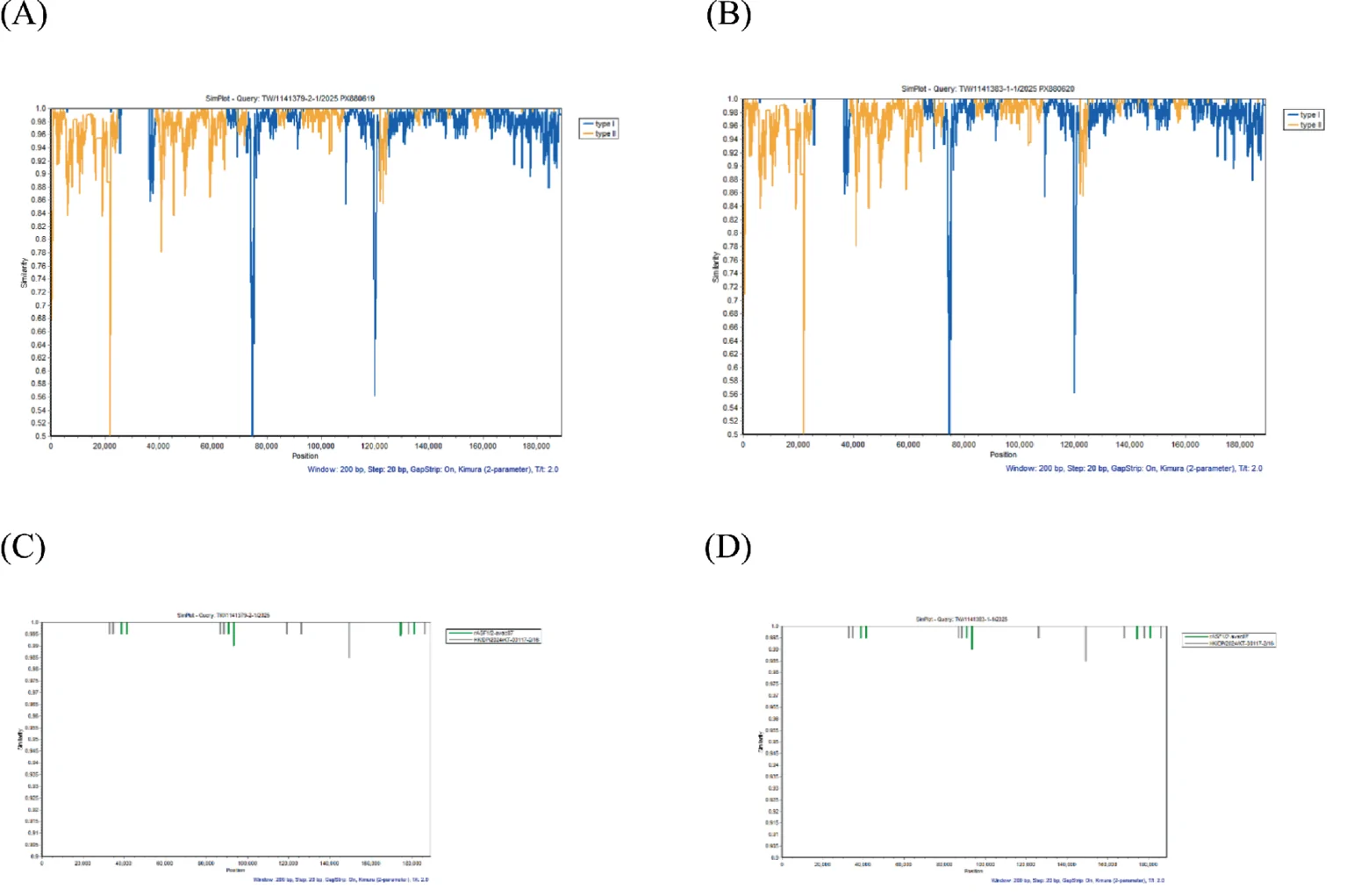
Recombinant analysis of isolates 1141379–2-1 and 1141383–1-1 performed using SimPlot software. **(A,B)** Recombination analysis of 1141379–2-1 and 1141383–1-1 identifying them as recombinants between genotypes I and II. **(C,D)** Comparison of the 1141379–2-1 and 1141383–1-1 isolates against rASFV/China and rASFV/Vietnam, evaluating the genetic profile of the 1141379 and 1141383 isolates.

The recombinant results from the SimPlot analysis demonstrated that both isolated viruses were rASFV (Figures 7A,B). Furthermore, both 1141379–2-1 and 1141383–1-1 were indistinguishable from rASFV/China or rASFV/Vietnam isolates (Figures 7C,D). The discordant genotype assignments based on the whole genome and the P72, P54, P30, and CD2v genes, together with their sequence identity to previously reported genotype I/II recombinant strains, indicate that the ASFV isolates from cases 1141379 (GenBank: PX880619) and 1141383 (GenBank: PX880620) are consistent with a genotype I/II recombinant lineage. This lineage is similar to those reported previously in China in 2022 and Vietnam in 2023 (4, 5), suggesting ongoing regional evolution and geographical expansion of these recombinant strains. Further analysis of the motif repeats in ASFV/TWN/2025, there found three repeats of I73-I329L intergenic region (IGR) tandem repeat (GAATATATAG) and was classified into IGR-II, was same as the rASFV/China or rASFV/Vietnam isolates.

## Discussion

Despite the persistent risk of ASF introduction via frequent international trade, large volume of economic activities, and cross-border business since August 2018, the domestic swine population in Taiwan remained free of the disease due to proactive border quarantine and surveillance. Given previous research, pork products carried by international travelers pose a high risk and are prioritized for border control in international airports and seaports (10). While airport testing frequently detected ASFV in seized pork products from ASF-affected regions (23, 24), the domestic swine industry remained uninfected until the current first ASF-infected case in 2025. This study documents the first confirmed ASF case on a domestic pig farm in central Taiwan. By characterizing this first ASF-infected farm, we illustrate the challenges of maintaining ASF-free status and provide critical virological evidence concerning the entry and spread of the virus in a previously unaffected region.

In the early phase of ASF pathogenesis, clinical signs alone are insufficient for reliable differential diagnosis (25), as lesions are non-specific and may be confused with other porcine hemorrhagic diseases such as CSF, highly pathogenic PRRS, or porcine dermatitis nephropathy syndrome. Therefore, effective molecular diagnosis and contact tracing rely on an early warning system based on abnormal clinical or production alerts. In this study, a total of two alerts were triggered through the national dead-pig monitoring system at rendering plants, enabling a rapid veterinary and regulatory response. This surveillance system was critical for timely initiating epidemiological investigation and differential diagnosis for ASF, thereby minimizing the risk of secondary outbreaks. Utilizing predefined mortality thresholds, authorities were able to identify an abnormal mortality surge at the index farm, which was initially suspected to be a case of respiratory infection (in this case, *Actinobacillus pleuropneumoniae*). Similar surveillance models have been reported for CSF surveillance in the Netherlands (26). Ultimately, this early detection enabled timely confirmation of a transboundary animal disease and underscores the value of strengthening such early warning systems for application in the Asia-Pacific.

Due to the substantial overlap in clinical signs among ASF and other major swine diseases, accurate diagnosis based solely on clinical observation is often challenging. Therefore, the availability of rapid and reliable differential diagnostic methods is of paramount importance for effective ASF prevention and control. Early and precise identification of the causative pathogen not only enables confirmation or exclusion of ASFV, but also guides decision-making regarding quarantine, movement restrictions, culling, and other emergency response measures. In this way, diagnostic capacity serves as a cornerstone of ASF preparedness by minimizing delays in response and preventing unnecessary economic losses caused by inappropriate interventions. During the ASFV outbreak in Taiwan, two alerts were generated by the early warning system but rapid differential diagnosis targeting ASFV, CSFV, PRRSV, and PCV2 was promptly conducted and the causative pathogen was confirmed. Based on these diagnostic results, emergency response protocols and epidemic prevention measures were immediately activated (27), enabling timely implementation of control strategies and substantially reducing the risk of ASFV transmission and spread within Taiwan’s swine industry. In this case, this further highlights the important of disease diagnosis in epidemic prevention.

Based on the pathological findings, rASFV/Taiwan exhibited high virulence, causing severe systemic hemorrhage, marked splenomegaly, and high mortality in experimentally infected pigs. The pathological manifestations were largely comparable to those reported for the recombinant ASFV strain identified in Vietnam (28, 29). Histopathological and immunohistochemical analyses demonstrated that rASFV/Taiwan was widely distributed in multiple organs, with viral antigen predominantly detected in cells of the monocyte–macrophage lineage, the principal target cells of ASFV. In addition, viral antigen was occasionally observed in vascular endothelial cells, suggesting endothelial involvement that may contribute to the widespread hemorrhagic lesions observed in infected animals.

As this outbreak primarily affected finishing pigs, a proportion of the animals were concurrently infected with PCV2 or PRRSV. However, quantitative PCR revealed relatively low viral loads of PCV2 and PRRSV, and characteristic pathological lesions associated with porcine circovirus-associated disease (PCVAD) or porcine respiratory disease complex (PRDC) were absent. These findings indicate that the affected pigs were likely in a subclinical stage of PCV2 or PRRSV infection rather than suffering from active clinical disease. Furthermore, comparison of ASFV genome copy numbers between PCV2-positive and PCV2-negative pigs showed no significant difference, suggesting that subclinical PCV2 infection did not enhance ASFV replication or disease severity. Collectively, these results indicate that the severe clinical outcomes observed in this outbreak were primarily attributable to infection with rASFV/Taiwan, rather than to concurrent subclinical infections with PCV2 or PRRSV.

In this study, multiple complementary diagnostic approaches were employed to confirm the ASFV detection in a domestic farm in Taiwan. Although pathological examination revealed gross lesions consistent with atypical ASF, definitive confirmation was carried out and achieved through WOAH-recognized standard virus isolation (Figure 5), combined with porcine erythrocyte HAD assay. Beyond initial validation via multi-gene qPCRs (P72, P54, P30, and CD2v), whole-genome sequencing and phylogenetic analysis validated that the isolated ASFV belongs to a recombinant genotype I/II strain. This molecular profile aligned with isolations reported in China (2022) and Vietnam (2023), providing evidence for the regional dissemination of emerging recombinant lineages.

Notably, the molecular identification of a genotype I/II recombinant excluded the introduction of vaccine-derived strains, chronic genotype I, or the typical genotype II strains previously widespread across Southeast Asia. Identification of this recombinant strain has direct implications for immediate prevention and control strategies. First, currently available commercial vaccines—which are designed to target genotype II—do not provide cross-protection against genotype I/II recombinants (9). Consequently, deploying or prioritizing emergency vaccination campaigns would be ineffective against this strain, shifting the immediate focus of regulatory authorities away from immunization and entirely toward strict biosecurity and stamping-out policies. Second, findings from this study indicate that clinical and pathological features alone are insufficient to distinguish between genotype II and genotype I/II infections due to their broadly overlapping field presentations. This diagnostic ambiguity emphasized the critical necessity of rapid, multiplex molecular surveillance at the borders and farm level to accurately identify circulating ASF variants.

In the literature, mortality in domestic pigs infected with ASF genotype II can reach up to 90–100% (25). Consequently, regulatory frameworks in countries like Vietnam in 2019, dictate that once a herd’s mortality rate exceeds a threshold of 50%, official post-mortem examinations and diagnostic sampling are triggered for laboratory confirmation (30, 31). However, current field realities present a much more nuanced picture. As ASFV continues to evolve and expand into new geographic areas across the Asia-Pacific region, emerging variants frequently cause lower mortality rates accompanied by more diverse clinical manifestations, such as reproductive failure, within affected herds (32). Consistent with this trend, in this research, the observed mortality rate on the affected farm was 35.2%. This lower mortality figure can be attributed to two distinct factors. First, from a regulatory standpoint, Taiwan’s active monitoring systems allowed for early detection; a significant proportion of the swine herd was likely still in the incubation period and was promptly depopulated before full clinical mortality could manifest. Second, from an epidemiological standpoint, lower field mortality can be influenced by producer behavior. For instance, outbreak investigations in the Dominican Republic have shown field mortality rates below 90–100%, driven in part by economic incentives for some producers to rapidly market or sell infected pigs before high-mortality from ASF occurs (33, 34). Ultimately, this landmark first ASF case demonstrates that a lower mortality rate does not exclude ASF. Instead, this figure should be regarded as a critical early warning signal, providing a useful field-based reference to prompt immediate diagnostic testing and accelerate the differentiation of ASF from other endemic hemorrhagic or respiratory conditions in future outbreaks.

Collectively, our findings underscore the limitations of relying solely on clinical and pathological assessments and reinforce the necessity of molecular diagnostics and whole-genome sequencing for accurate strain characterization. In addition, the detection of this recombinant strain in Taiwan suggests ongoing geographic expansion of the genotype I/II lineage, following prior reports in China (4), Hong Kong (35), Vietnam (5, 29), and Russia (36). This pattern highlights the transboundary nature of ASF and warrants heightened vigilance, serving as a warning signal for the Asia-Pacific region. Continued surveillance and regional collaboration remain essential for future ASF preparedness.

Although the molecular profile is confirmed, the point of introduction from the border to the domestic farm was undetermined due to logistical difficulties. Determining the specific transmission pathways of this breach is essential for refining future prevention strategies. Plausible pathways include the legal or illegal entry of ASFV-contaminated pork products, swill feeding practices in farming, and/or indirect transmission via contaminated fomites. In response to these potential risks, the authority has strengthened the biosecurity framework, including a policy revision to fully prohibit feeding swill and food garbage to domestic swine by 2027, with a one-year transition period to support current swine producer compliance.

Following the ASF confirmation, rapid diagnostic procedures enabled the immediate implementation of control measures. A rigorous containment strategy was executed, including the depopulation of affected pigs and the establishment of a 3-km control zone surrounding the affected premises. Concurrently, a nationwide swine movement standstill was immediately enforced for 15 days following the case confirmation. This public health intervention, coordinated across different governmental sectors and supported by public–private partnerships, facilitated the implementation of an intensive nationwide surveillance program. The temporary disruption of the swine supply chain created a critical window for surveillance teams to conduct a risk assessment across the country. By the end of the standstill period, no additional cases had been detected. Surveillance activities, including clinical inspections of 5,442 commercial swine farms and 434 registered swill-feeding operations, identified no additional suspected ASF cases, confirming that the potential outbreak was successfully contained within the case farm. The outcome demonstrates that rapid molecular diagnosis combined with decisive large-scale movement control can effectively interrupt ASFV transmission, highlighting the necessity of coordinated response capacity in previously ASF-free regions like Taiwan.

## Conclusion

At the national level, this ASFV case highlights the importance of a multi-layered biosecurity strategy. The effectiveness of the early response relied on at least three key components: first, the deployment of integrated digital passive surveillance systems across the swine supply chain, particularly mortality alerts from rendering plants; second, the rapid application of molecular diagnostics (PCR, virus isolation, and whole-genome sequencing), which enabled definitive characterization of a recombinant ASFV genotype I/II strain within 48 h of sample collection and supported an immediate evidence-based response by the central government; and third, the implementation of coordinated control measures across multiple government sectors. Rapid supply chain interventions, including temporary standstill measures to interrupt potential secondary transmission, together with effective inter-agency communication and general public compliance with movement controls, collectively helped contain the outbreak within the affected farm and prevent a large-scale ASF epidemic in Taiwan.

In conclusion, Taiwan’s experience in response to its first ASF case demonstrated that a resilient, multi-layered biosecurity framework can offset border control vulnerabilities, provided that domestic emergency response systems are highly digitized, rapidly activated, and centrally coordinated. Success relied on cross-sector collaboration among multiple government agencies and systematic surveillance to evaluate and exclude alternative infection pathways, effectively preventing further viral introduction.

## Data Availability

The data presented in the study are deposited in the NCBI GenBank repository, accession numbers PX880619 and PX880620.
